# Associations Between Gambling Motives and More Severe Gambling Disorder – Does Gender Matter?

**DOI:** 10.1007/s10899-025-10470-3

**Published:** 2026-02-23

**Authors:** Larissa Schwarzkopf, Bernhard Rosenboom, Bianca Pitzschel, Andreas Manuel Bickl

**Affiliations:** 1 Institute for Medical Information Processing, Biometry, and Epidemiology - IBE, LMU Munich, Munich, Germany; 2 Pettenkofer School of Public Health, Munich, Germany; 3 IFT Institut für Therapieforschung, Centre for Mental Health and Addiction Research, Munich, Germany

**Keywords:** Pathological gambling, Problem gambling, Disease progression, Motivation, Sex factors, Observational study

## Abstract

Gambling Disorder (GD) is a behavioural addiction with complex, detrimental psychological, economic, and social consequences. Previous research has examined gender differences in the prevalence of distinct gambling motives and has linked gambling motives to at-risk behaviour. Whether motives affect probabilities of transition to higher GD severity levels differently in males and females remains unclear. Using an online self-test, we assessed the prevalence distinct GD severity levels according to the DSM-5 criteria and gambling motives with items addressing domains of the “Gambling Motives Questionnaire – Financial” (GFQ-F). Associations between motives and GD severity at three transition points (no GD to mild, mild to moderate, moderate to severe GD) were analysed using age-adjusted logistic adjacent category regression models. Interaction terms (motive*gender) were examined to detect gender-specific “effects”. Furthermore gender-stratified models were run. No significant gender*motive interactions emerged. In the total sample, a transition to a higher GD severity level was predicted by: “(to) Forget worries”, “When depressed,” and “(to) Earn money” at each transition point; “Thrill” and “Stress” at the first two points, and “Boredom” at the first point. In males, the results were nearly identical. In females only “Thrill”, “Boredom”, and “(to) Earn money” were predictive at the first transition point. Given the prevalence of coping motives in females and the consistent detrimental “effect” of coping and financial motives in males, these motives should be prioritized in treatment and support offers for both genders. For prevention, strategies targeting enhancement motives may be particularly effective.

**Supplementary Information:**

The online version contains supplementary material available at https://doi.org/10.1007/s10899-025-10470-3.

## Background

Gambling, defined as wagering money or something else of value on a random event with the aim of gaining more (Potenza et al., 2002), constitutes a common leisure activity. A recent meta-analysis showed that almost half (46%) of adults globally had engaged in gambling during the previous 12 months, with males participating more often than females (49% vs. 37%) (Tran et al., 2024). Furthermore, the study indicated that about one in seven males gambled to a potentially detrimental extent (engagement in risky gambling: 12%; engagement in problematic gambling 2%), as compared to one in fifteen females (engagement in risky gambling: 6%; engagement in problematic gambling 1%) (Tran et al., 2024).

These findings suggest that males face a higher risk of developing detrimental (i.e., at risk or disordered) gambling behaviour than females, and is supported by meta-analytic evidence determining male gender as a robust predictor for engagement in (at least) risky gambling behaviour (Allami et al., 2021). This gender difference might partly be attributed to greater risk-taking and higher social anxiety among males, which, in combination with comparatively lower levels of impulsive coping, may facilitate more frequent gambling engagement (Wong et al., 2013). Consistent with this notion, sensation seeking (Donati et al., 2013) and trait impulsivity (Choi & Kim, 2021; González-Ortega et al., 2013) were determined to be key predictors for severity of gambling disorder (GD) in males, whereas emotional distress and mood-related vulnerability (e.g., depressiveness, low self-esteem, anxiety) were found to co-occur more frequently with gambling-related issues in females (Choi & Kim, 2021; González-Ortega et al., 2013; Lloyd et al., 2010). Acknowledging that gambling engagement and detrimental gambling are intertwined with distinct, potentially gender-specific personality traits, it is plausible that gambling motives differ between males and females. The “Gambling Motives Questionnaire ꟷ Financial” (GMQ-F) is a widely used tool which assesses four motivational dimensions of gambling ꟷ coping (“to reduce negative emotions”), enhancement (“to augment positive emotions”), social (“to enhance affiliation with others”), and financial (“to earn money”) motives ꟷ with strong measurement invariance across sex (Schellenberg et al., 2015).

Evidence on the gender-specific relevance of gambling motives remains inconclusive. While some studies indicate that males gamble more often for enhancement or monetary gains (Echeburua et al., 2011; Lloyd et al., 2010; Walker et al., 2005) and females gamble more often for coping (Ledgerwood & Petry, 2006; Stewart et al., 2008; Wenzel & Dahl, 2008), other research demonstrates similar motive patterns across genders (Clarke et al., 2007; Clarke, 2008; Hagfors et al., 2022; Sundqvist et al., 2016). These discrepancies might be explained by differences between study samples (e.g., clinical vs. population samples), as gender differences in social, coping, and enhancement (but not financial) motives decrease as gambling behaviour becomes more detrimental (Flack & Stevens, 2018).

In contrast, the association between gambling motives and detrimental gambling behaviour is understood quite well. The original GMQ-F study demonstrated a direct association of coping motives with severity of GD, while the effects of enhancement, social, and financial motives were mediated by gambling frequency (Schellenberg et al., 2015). A recent meta-analysis confirmed coping motives as a strong predictor of problematic gambling behaviour, while enhancement motives presented a medium sized and social as well as financial motives a small association (Allami et al., 2025).

Much of this evidence is derived from samples with a male majority, making generalizability to female populations unclear (Hing et al., 2016; McCarthy et al., 2019). Thus, despite consensus that distinct motives for gambling engagement are linked to different risks for developing detrimental gambling behaviour, it is unclear whether the negative impact of gambling motives differs by gender. Given this background, our study aims to

- assess the gambling motives of males and females exhibiting various GD severity levels.
- evaluate the association of gambling motives with transitions to higher GD severity levels within a (pre-)clinical sample of individuals who consider themselves at risk for detrimental gambling behaviour.

## Methods

### Participants

Data for this study were obtained from the online self-assessment tool “*Verspiel nicht dein Leben”* (“Don’t Gamble Away Your Life”; VNDL), hosted by the Bavarian Coordination Centre for Gambling Issues (Landesstelle Glücksspielsucht in Bayern, LSG; https://www.verspiel-nicht-dein-leben.de/selbsttest). This tool is designed to assist individuals in evaluating their gambling behaviour by providing personalised feedback, and, where appropriate, directing them to relevant support services. Therefore, individuals with (more pronounced) detrimental gambling behaviour are over-represented compared to the total German resident population.

We included all unique, non-occupational VNDL-tests completed between 1 July 2021, and 30 June 2024. This period corresponds to the first three years following the implementation of the revised German State Treaty on Gambling (GlüStV 2021), which legalised online gambling that had previously been prohibited. Accordingly, variations in response patterns before and after this regulatory change were anticipated.

We only analysed VNDL-tests with complete self-reported data on age, self-attributed gender, gambling motives, and gambling-related problems (*n* = 2,192). The sample was restricted to individuals aged 18 to 70 years (*n* = 2,105). Due to the small number of participants reporting a non-cisgender (German: “*divers*”) identity (*n* = 51), they were excluded, yielding a final sample of 2,054 participants. As gender assigned at birth may be more strongly associated with detrimental gambling behaviour than adult gender identity (Malkin & Stacey, 2024), the exclusion of participants with diverse gender identities was considered methodologically justifiable despite their elevated risk for coping-related gambling (Lee & Grubbs, 2023).

## Measures

The VNDL captures (a) the types of gambling activities one engaged in, (b) self-perceived gambling-related problems, and (c) gambling motives. Gambling-related problems over the past 12 months were assessed using the DSM-5-adapted Stinchfield criteria (excluding items related to illegal activities) ꟷ a validated instrument for detecting GD (Stinchfield, 2003). The sum score of nine dummy-coded problem indicators (criteria) indicates no GD (0–3 criteria) and mild (4–5 criteria), moderate (6–7 criteria), or severe (8–9 criteria) GD (American Psychiatric, 2022).

Gambling motives were assessed using a 10-item list based on the GMQ-F which covered *coping *(“(to) Forget worries”, “Stress”, “When depressed”, “Boredom” [as a reason for inclusion see: (Myrseth & Notelaers, 2017)); *enhancement* (“Fun”, “Thrill”);* social* (“Gambling friends”, “Socializing”); and *financial* (“(to) Earn money”) motives]. Additionally, “Habit” was included for a sensitivity analysis due to the ongoing debate regarding whether habit is an independent motivational domain or a behavioural pattern developed through repeated gambling and driven by other motives (Ferrari et al., 2022; Mizerski et al., 2013; van Timmeren & Clark, 2025).

### Statistical Analysis

We first examined the mean age (± standard deviation/SD), prevalence of individual gambling motives, and distributions of GD severity levels ꟷ both overall and stratified by gender. Additionally, we examined the gender-specific prevalence of gambling motives in the total sample and across GD severity levels. Gender differences were evaluated using Mann-Whitney-U tests for age and χ²-tests for presence of motives and GD severity. All p-values were calculated with the Bonferroni–Holm method.

To examine the association between motives and increased GD severity, we first assessed multicollinearity among motives using variance inflation factors (VIF; cut-off: VIF > 5) (Kim, 2019) and Pearson correlation coefficients (cut-off: *r* >.6) (Singh, 2023). In cases of multicollinearity, the motive with greater explanatory power would have been retained. Associations were then analysed via ordered logistic regression (OLR) to account for the ordinal nature of the dependent variable “GD severity” (Fullerton, 2009). We applied the adjacent-category (ACAT) approach, which estimates log odds for being assigned to the next higher GD severity level versus the current level (i.e., mild vs. no GD, moderate vs. mild, severe vs. moderate) (Bürkner & Vuorre, 2019; Fullerton, 2015). This method allows for non-parallel slopes and relaxes the proportional odds assumption, offering flexibility and interpretability (Bürkner & Vuorre, 2019; Fullerton, 2015). For ease of interpretation, we refer to these log odds as “transition probabilities”, although they reflect cross-sectional, between-subject comparisons rather than longitudinal, within-subject changes.

As we aimed to develop a parsimonious, interpretable model rather than a highly accurate predictive model, we utilised stepwise variable selection (Breiman, 2001; Chowdhury & Turin, 2020). Model selection began with univariate OLRs with age and gender as default covariates. Motives with at least one significant transition probability (*p* <.05) were included in a multivariate stepwise model, again controlling for age and gender. Gender*motive interactions were included to account for potential gender-specific "effects". Motives showing significance at no more than one transition point were further evaluated via likelihood ratio tests (LRT), Akaike information criterion (AIC), and Bayesian information criterion (BIC) to determine the most parsimonious model. Gender-stratified models with age included by default were also examined. To check the robustness of our models we performed two sensitivity analyses: one included “habit” as an additional motive for gambling engagement and the other included all possible motive*age interactions.

The statistical analyses were conducted using RStudio 2024.04.2 + 764 (Posit Software, PBC, formerly RStudio, PBC, Boston, MA), using the VGAM package for the OLR models (Yee, 2010) at an exploratory alpha-level of 5%.

## Results

### Sample Description

Males made up 84.2% (*n* = 2,054) of participants. The mean age of the total sample was 33.8 years (SD = ±12.1). Males were, on average, significantly younger than females (mean = 32.6 (±11.6) years vs. mean = 39.9 (±12.7) years; *p* <.001). About one in four participants qualified for mild GD (23.8%; males: 24.5%; females: 2.1%), about one in five qualified for moderate GD (21.0%; males: 20.8%; females: 22.2%), and about one in eight qualified for severe GD (13.2%; males: 12.8%; females: 15.7%). The distribution of GD severity levels did not differ significantly across genders.

**Fig. 1:**
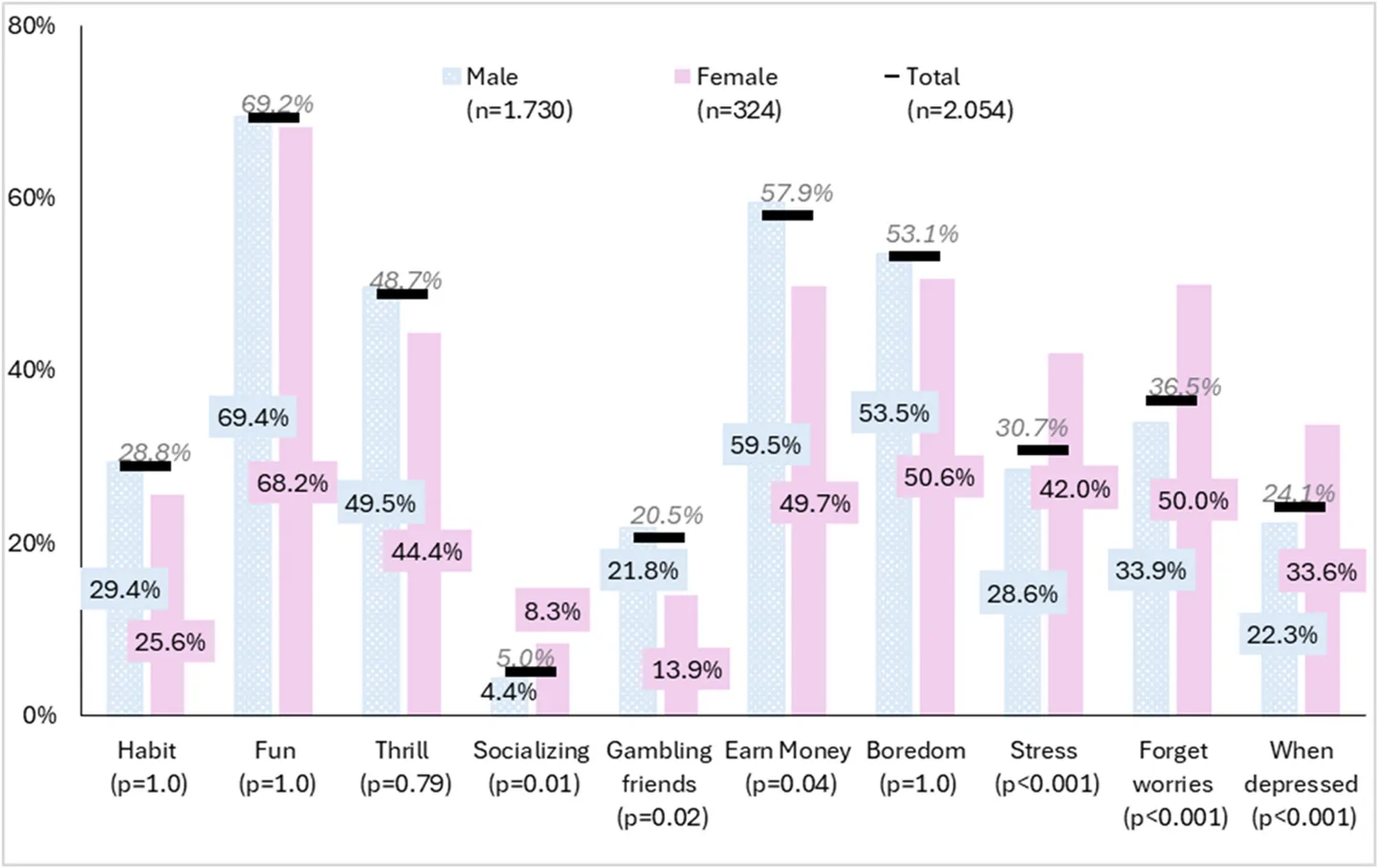
Gambling motives among males and females

Males most often reported the motives “Fun” (69.4%), “(to) Earn money” (59.5%), and “Boredom” (53.5%). Females reported “Fun” (68.2%) most often, followed by “Boredom” (50.6%), “(to) Forget worries” (50.0%), and “(to) Earn money” (49.7%) with almost the same prevalence (see Fig. 1). Compared to males, females reported “(to) Earn money” (*p* =.04) and “Gambling friends” (13.9% vs. 21.8%; *p* =.02) motives less often, but “Socializing” (8.3% vs. 4.4%; *p* =.01) and coping motives [“(to) Forget worries” (50.0% vs. 33.9%; *p* <.001); “Stress” (42.0% vs. 28.6%; *p* <.001), “When Depressed” (33.6% vs. 22.3%; *p* <.001)] more often.

GD = Gambling disorder.

The share of female participants was similar across all GD severity levels (no GD: 15.8%; mild GD: 13.3%; moderate GD: 16.6%; severe GD: 18.8% |) and within each GD severity level females had a higher average age than males. We found no clear trends in motive prevalence and GD severity level, but males and females with GD reported “Thrill”, “Stress”, “Boredom”, When Depressed”, “(to) Forget”, and “(to) Earn Money” more often than participants with no GD. “Socializing” was by far the most important motive for participants who qualified for severe GD (see Figure S1).

Comparing females to males at each GD severity level revealed the following differences (see Figure S1): females who presented with no GD reported most coping motives [“Stress”, “(to) Forget worries”, and “When Depressed”] and “Socializing” more often than males, but “Thrill” and “(to) Earn Money” less often. Females who qualified for mild GD also reported these three coping motives more often, but “Gambling friends” less often. Females who qualified for moderate GD reported “When Depressed” more often. No gender-differences emerged in participants who qualified for severe GD.

### Association of Gambling with Transition To Higher Severity Levels of Gambling Disorder

#### Total Population

VIF-values (highest: 1.67 (“Stress”)) and correlation coefficients (highest:.57 (“Stress”|”When Depressed”)) did not indicate multicollinearity. For the total population “Thrill”, “Stress”, “(to) Forget Worries”, “When Depressed”, “(to) Earn Money”, “Boredom”, and “Socializing” as well as the interaction female*“(to) Earn Money” were selected for the multivariate model. During the post-hoc checks, the interaction and “Socializing” were dropped because they did not substantially improve model fit (Full model: AIC = 4.534, BIC = 4.720; Reduced Model: AIC = 4.537, BIC = 4.689; LRT: *p* =.054).

“(To) Forget Worries”, “When Depressed”, and “(to) Earn Money” were predictive of higher GD severity level at each transition point; “Thrill” and “Stress” were predictive at the first (no GD to mild) and second (mild to moderate) transition point; and “Boredom” was predictive at the first transition point. Female gender was not associated with any transition point. All significant associations indicated an increased transition probability when the respective motive was present (see Table 1).

**Table 1:** Predictors for transition to higher severity levels of gambling disorder

	Est.	SE	z value	*p* value	OR	95%- CI	
*(Intercept): no GD → mild*	*−1.89*	*0.23*	*−8.32*	*0.00*	*0.15*	*0.10*	*0.24*
*(Intercept): mild → moderate*	*− 0.42*	*0.27*	*−1.53*	*0.13*	*0.66*	*0.39*	*1.12*
*(Intercept): moderate→ severe*	*−1.23*	*0.34*	*−3.63*	*0.00*	*0.29*	*0.15*	*0.57*
*Female: no GD → mild*	*− 0.25*	*0.18*	*−1.40*	*0.16*	*0.78*	*0.54*	*1.11*
*Female: mild → moderate*	*0.25*	*0.20*	*1.25*	*0.21*	*1.28*	*0.87*	*1.89*
*Female moderate → severe*	*− 0.02*	*0.21*	*− 0.09*	*0.93*	*0.98*	*0.65*	*1.49*
*Age: no GD → mild*	*0.00*	*0.01*	*− 0.83*	*0.41*	*1.00*	*0.99*	*1.01*
*Age: mild → moderate*	*− 0.02*	*0.01*	*−3.02*	*0.00*	*0.98*	*0.97*	*0.99*
*Age: moderate → severe*	*− 0.01*	*0.01*	*− 0.71*	*0.48*	*1.00*	*0.98*	*1.01*
Thrill: no GD → mild	0.44	0.12	3.52	**< 0.001**	1.55	1.21	1.98
Thrill: mild → moderate	0.30	0.14	2.15	**0.03**	1.35	1.03	1.78
Thrill: moderate → severe	− 0.13	0.17	− 0.78	0.43	0.88	0.63	1.22
Earn money: no GD → mild	0.89	0.13	7.09	**< 0.001**	2.45	1.91	3.13
Earn money: mild → moderate	0.43	0.15	2.92	**< 0.001**	1.53	1.15	2.04
Earn money: moderate → severe	0.36	0.18	2.04	**0.04**	1.43	1.01	2.03
Boredom: no GD → mild	0.88	0.13	7.03	**< 0.001**	2.41	1.89	3.08
Boredom: mild → moderate	− 0.11	0.14	− 0.75	0.45	0.90	0.68	1.19
Boredom: moderate → severe	− 0.20	0.17	−1.15	0.25	0.82	0.59	1.15
Stress: no GD → mild	0.86	0.18	4.89	**< 0.001**	2.36	1.67	3.34
Stress: mild → moderate	0.36	0.17	2.13	**0.03**	1.44	1.03	2.01
Stress: moderate → severe	0.08	0.20	0.40	0.69	1.08	0.73	1.60
Forget worries: no GD → mild	0.63	0.15	4.10	**< 0.001**	1.88	1.39	2.54
Forget worries: mild → moderate	0.48	0.16	3.06	**< 0.001**	1.62	1.19	2.20
Forget worries: moderate → severe	0.85	0.19	4.40	**< 0.001**	2.34	1.60	3.43
When depressed: no GD → mild	0.52	0.20	2.62	**0.01**	1.68	1.14	2.49
When depressed: mild → moderate	0.48	0.18	2.71	**0.01**	1.62	1.14	2.30
When depressed: moderate → severe	0.63	0.19	3.31	**< 0.001**	1.88	1.29	2.74

bold values are significant;

CI = 95% confidence interval, Est = Estimate; OR = Odds Ratio, SE = Standard error

In the first sensitivity analysis, “Habit” was included in the multivariate model, which improved goodness of fit compared to the initial model (sensitivity analysis 1: AIC = 4,479, BIC = 4,648; main analysis: AIC = 4,537, BIC = 4,689; LRT: p <.001). “Habit” was predictive at each transition point. Otherwise, the results of the main analysis were by and large confirmed (see Table S1). In the second sensitivity analysis, the interaction age*“(to) Forget Worries ” was initially included but was subsequently dropped from the multivariate model because it did not substantially improve model fit according to post hoc tests (sensitivity analysis 2: AIC = 4,534, BIC = 4,720; main analysis: AIC = 4,537, BIC = 4,689; LRT: *p* =.06).

## Male Population

For males, “Thrill”, “Stress”, “Boredom”, “(to) Forget Worries”, “When Depressed”, and “(to) Earn Money” were selected for the multivariate model. Post hoc checks supported the initial selection.

While “(to) Forget Worries”, “When Depressed”, and “(to) Earn Money” were predictive at each transition point, “Thrill” was predictive at the first and the second transition point, and “Stress” as well as “Boredom” were predictive only at the first transition point (see Table 2). All significant associations indicated an increased transition probability given the presence of the motive.

**Table 2:** Predictors for transition to higher severity levels of gambling disorder in males

	Est.	SE	z value	*p*-value	OR	95%- CI	
*(Intercept): no GD → mild*	*−1.74*	*0.24*	*−7.12*	*< 0.001*	*0.17*	*0.11*	*0.28*
*(Intercept): mild → moderate*	*− 0.39*	*0.29*	*−1.32*	*0.19*	*0.68*	*0.38*	*1.21*
*(Intercept): moderate → severe*	*−1.62*	*0.38*	*−4.28*	*< 0.001*	*0.20*	*0.09*	*0.41*
*Age: no GD → mild*	*− 0.01*	*0.01*	*− 0.94*	*0.35*	*0.99*	*0.98*	*1.01*
*Age: mild → moderate*	*− 0.02*	*0.01*	*−2.65*	*0.01*	*0.98*	*0.97*	*1.00*
*Age: moderate → severe*	*< 0.001*	*0.01*	*0.08*	*0.93*	*1.00*	*0.98*	*1.02*
Thrill: no GD → mild	0.39	0.13	2.92	**< 0.001**	1.47	1.14	1.91
Thrill: mild → moderate	0.32	0.15	2.13	**0.03**	1.38	1.03	1.86
Thrill: moderate → severe	− 0.17	0.19	− 0.89	0.37	0.85	0.58	1.22
Stress: no GD → mild	0.89	0.19	4.58	**< 0.001**	2.42	1.66	3.54
Stress: mild → moderate	0.32	0.19	1.75	0.08	1.38	0.96	1.99
Stress: moderate → severe	0.13	0.22	0.61	0.54	1.14	0.74	1.75
Forget worries: no GD → mild	0.66	0.17	3.96	**< 0.001**	1.94	1.40	2.68
Forget worries: mild → moderate	0.40	0.17	2.35	**0.02**	1.48	1.07	2.07
Forget worries: moderate → severe	0.84	0.21	4.02	**< 0.001**	2.32	1.54	3.50
Earn money: no GD → mild	0.77	0.13	5.75	**< 0.001**	2.17	1.66	2.82
Earn money: mild → moderate	0.41	0.16	2.62	**0.01**	1.51	1.11	2.05
Earn money: moderate → severe	0.45	0.20	2.25	**0.02**	1.57	1.06	2.31
Boredom: no GD → mild	0.81	0.13	6.02	**< 0.001**	2.24	1.73	2.92
Boredom: mild → moderate	− 0.09	0.16	− 0.57	0.57	0.92	0.67	1.24
Boredom: moderate → severe	− 0.06	0.19	− 0.30	0.76	0.94	0.65	1.38
When depressed: no GD → mild	0.53	0.22	2.34	**0.02**	1.69	1.09	2.63
When depressed: mild → moderate	0.47	0.2	2.37	**0.02**	1.60	1.08	2.36
When depressed: moderate → severe	0.75	0.21	3.52	**< 0.001**	2.12	1.4	3.22

bold values are significant;

CI = 95% confidence interval, OR = Odds Ratio, SE = Standard error

In sensitivity analysis 1, “Habit” was included in the final model because it was predictive at the first and second transition point. Otherwise, the results resembled those of the main analysis (see Table S2). Sensitivity analysis 2 did not reveal a relevant age*motive interaction.

### Female Population

For females, “Thrill”, “Stress”, “Boredom”, “(to) Forget Worries”, “When Depressed”, and “(to) Earn Money” were selected for the multivariate model. Post hoc checks supported the initial selection.

Among females, “(to) Earn Money”, “Thrill”, and “Boredom” were predictive at the first transition point. “(To) Forget Worries” was predictive at the second transition point (See Table 3).

**Table 3:** Predictors for transition to higher severity levels of gambling disorder in females

	Est.	SE	z value	*p*-value	OR	95%- CI	
*(Intercept)*: no GD *→ mild*	*−3.16*	*0.70*	*−4.49*	*0.00*	*0.04*	*0.01*	*0.17*
*(Intercept): mild → moderate*	*−0.38*	*0.81*	*−0.46*	*0.64*	*0.69*	*0.14*	*3.37*
*(Intercept): moderate → severe*	*0.78*	*0.86*	*0.92*	*0.36*	*2.19*	*0.41*	*11.71*
*Age*: no GD *→ mild*	*< 0.001*	*0.01*	*0.10*	*0.92*	*1.00*	*0.98*	*1.03*
*Age: mild → moderate*	*−0.02*	*0.02*	*−1.42*	*0.16*	*0.98*	*0.95*	*1.01*
*Age: moderate → severe*	*−0.03*	*0.02*	*−1.80*	*0.07*	*0.97*	*0.94*	*1.00*
Thrill: no GD → mild	0.91	0.37	2.45	**0.01**	2.49	1.20	5.18
Thrill: mild → moderate	−0.02	0.39	−0.04	0.96	0.98	0.46	2.10
Thrill: moderate→ severe	0.18	0.42	0.43	0.67	1.19	0.53	2.70
Stress: no GD → mild	0.77	0.45	1.71	0.09	2.15	0.90	5.16
Stress: mild → moderate	0.47	0.46	1.03	0.30	1.60	0.65	3.94
Stress: moderate → severe	−0.14	0.51	−0.28	0.78	0.87	0.32	2.36
Forget worries: no GD → mild	0.50	0.43	1.17	0.24	1.65	0.71	3.79
Forget worries: mild → moderate	1.14	0.46	2.46	**0.01**	3.14	1.26	7.78
Forget worries: moderate → severe	0.57	0.54	1.05	0.29	1.77	0.61	5.16
Earn money: no GD → mild	1.79	0.38	4.69	**< 0.001**	5.99	2.84	12.67
Earn money: mild → moderate	0.49	0.39	1.24	0.22	1.63	0.75	3.51
Earn money: moderate → severe	−0.11	0.40	−0.28	0.78	0.89	0.41	1.95
Boredom: no GD → mild	1.42	0.36	3.91	**< 0.001**	4.12	2.03	8.38
Boredom: mild → moderate	−0.29	0.40	−0.72	0.47	0.75	0.34	1.64
Boredom: moderate → severe	−0.70	0.40	−1.78	0.08	0.49	0.23	1.07
When depressed: no GD → mild	0.49	0.45	1.09	0.28	1.63	0.68	3.90
When depressed: mild → moderate	0.65	0.42	1.53	0.13	1.91	0.83	4.39
When depressed: moderate → severe	0.08	0.45	0.19	0.85	1.09	0.45	2.60

bold values are significant;

CI = 95% confidence interval, OR = Odds Ratio, SE = Standard error

In sensitivity analysis 1, “Habit” was included in the final model. It was associated with an increased transition probability at the last transition point. “Boredom” became predictive at the last transition point but with a mitigating effect. Otherwise, the results of sensitivity analysis 1 resembled those of the main analysis (see Table S4). Sensitivity analysis 2 did not reveal a relevant age*motive interaction.

## Discussion

This study examined the association between gambling motives and the probability of transition to more advanced GD severity levels with a focus on gender-specific mechanisms in a (pre-clinical) sample of individuals considering themselves at risk for detrimental gambling behaviour. The results show that males and females exhibit somewhat different gambling motives and these differences diminish at more advanced GD severity levels. However, no gender-specific “effects” of specific motives on transition probabilities were found. This may be due to the low number of female participants at advanced GD severity levels. Within the total sample, the range of motives that were predictive at the transition point from no GD to mild GD was broader than the range of predictive motives at the subsequent transition points. “(To) Forget Worries”, “When Depressed”, and “(to) Earn Money” were consistently predictive across the total sample and among males, whereas “Thrill”, “Stress”, and “Boredom” were only predictive at the first transition(s). No motive emerged as a new predictor at more advanced transition points.

Neither the whole sample nor the gender-stratified analyses revealed any associations between social motives and transition probabilities. This aligns with the mixed evidence regarding their directional impact and a generally weak association with detrimental gambling behaviour, as reported in a recent meta-analysis (Allami et al., 2025). Those authors suggested that the inconclusive findings may partly reflect the often-overlooked factor of whether individuals gamble within a social environment where others gamble as well. Another recent study indicated that social motives may only have a detrimental association when driven by social deficits (e.g., loneliness, poor social connectedness) (Floyd et al., 2025), which may be the case at more advanced GD severity levels. This fits with our finding that “Socializing” was most often reported by participants who qualified for severe GD (compared to those qualifying for lower GD severity levels) by far, while there was little variation in reports of “Gambling friends” across GD severity levels.

We found no link between “Fun” and transition probabilities, which agrees with the meta-analysis mentioned above (Allami et al., 2025). Nevertheless, in each GD severity level subgroup, “Fun” was among the three most frequently reported motives. This supports the observation that “fun-driven” gambling is not inherently risky but may mask the emergence of escalating gambling behaviours as people engaging in “fun-driven” gambling tend to underestimate their risk for and overlook early signs of detrimental gambling behaviour (Hing et al., 2015). In both genders, “Thrill”, the second enhancement motive, was not predictive at the last transition point (moderate to severe GD) but at both earlier transition points. This finding contributes to the on-going scientific discussion of whether a sensation-seeking personality is an independent risk factor for developing detrimental gambling behaviour (Canale et al., 2015) or if the negative impact of sensation-seeking comes from an impulsive personality trait (Hodgins & Holub, 2015) and high levels of problem externalization (Tani et al., 2020).

Notably, sensation-seeking in people who exhibit detrimental gambling behaviour may not stem solely from a desire for thrilling experiences, but could also indicate heightened “boredom susceptibility” (Fortune & Goodie, 2010). In fact, boredom was identified as the second most important predictor of detrimental gambling behaviour in the meta-analysis above (Allami et al., 2025) ꟷ but with substantial between-study heterogeneity. This variability may be due to differing definitions of boredom; specifically, “boredom susceptibility” (an enhancement-related trait marked by a low tolerance for boredom and a tendency to seek stimulation) and “boredom proneness” (a coping-related trait characterized by frequent feelings of boredom linked to inner emptiness). Evidence suggests that “boredom proneness”, rather than “boredom susceptibility”, triggers GD (Blaszczynski et al., 1990; Yakobi & Danckert, 2021). In our study, “Boredom” was predictive at the transition point from no GD to mild GD in both genders, but not at subsequent transitions points. This might substantiate its predominant definition of “boredom susceptibility”. Given that “boredom proneness” plays a key role in habit formation (Blaszczynski et al., 1990; Emir et al., 2025; Tagliaferri et al., 2025), it is plausible that “Habit” ꟷ which was predictive at each transition point according to our sensitivity analysis ꟷ indeed reflects some aspects of “boredom proneness”.

Among the remaining coping motives, “(to) Forget Worries” and “When Depressed” were consistently linked to increased transition probabilities in males. This supports the fundamental role of escapism and dysfunctional coping as established risk factors (Alaba-Ekpo et al., 2024; Allami et al., 2025; Neophytou et al., 2023). Contrastingly, no such association was found in females. This may be explained by the consistently higher prevalence of all coping motives and less variation in motive prevalence across GD severity levels among females in our study. This high prevalence might underpin the relevance of mood-regulating escapist motives for females who gamble, although they do not predict transition to more severe GD.

In contrast, gambling to relieve “Stress” ꟷ another coping motive ꟷ was only predictive at the first transition point in males, which fits with its acknowledged pronounced relevance in males (Wong et al., 2013). It can be argued that individuals who gamble to forget worries or to manage depression may present marked psycho-emotional vulnerability and may be particularly prone to persistent mental health issues (Blaszczynski & Nower, 2002; Ferro et al., 2024), whereas those who gamble for stress relief might tend to respond to specific, short-term stressors (Lloyd et al., 2010). The association of stress with detrimental gambling behaviour is, hence, apparently mediated through high levels of maladaptive coping (Caudwell et al., 2025), with brooding rumination as a sub-type of maladaptive coping being more likely in individuals with severe GD (Krause et al., 2018). Although mood-regulating escapist motives are more common in females, recent evidence suggests that males tend to demonstrate greater maladaptive coping patterns and impaired emotional regulation capacities (Girone et al., 2024; Neophytou, et al., 2023; Wong et al., 2013). Thus, it is plausible that, particularly in males, the coping-related aspects of forgetting and escaping from worries absorb the effect of stress relief at more advanced GD severity levels.

Finally, we identified financial motives as consistent predictors at each transition point in males and as a predictor at the first transition point in females. This partially contrasts with the previously cited meta-analysis (Allami et al., 2025) that suggested a minor role for “winning money”. This discrepancy may be due to different connotations of “winning” and “earning” money. The latter term encompasses not only direct monetary gains ꟷ which may be more relevant at the transition point from no GD to mild GD ꟷ but also the pursuit of “chasing losses”, a behaviour that is more prominent in later, more advanced stages of GD (Ciccarelli et al., 2019).

### Strengths and Limitations

This study’s findings should be interpreted with several caveats in mind ꟷ in addition to the small number of female participants acknowledged above. First, due to the cross - sectional design, we cannot determine how gambling motives evolve within an individual over time. Second, we deliberately did not include problem-causing gambling activities in our models to avoid over-adjustment. Consequently, observed differences between males and females, or lack thereof, may be influenced by the inclusion of different subgroups of people who gamble (e.g., those with engagement in electronic gaming machines, casino games, sports betting). There is consensus that certain gambling activities carry higher risks than others (Mazar et al., 2020), therefore, the type of gambling activity may affect transition probabilities. Presuming that individuals select gambling activities aligned with their intrinsic motives, we consider the exclusion of gambling activity types justified. Third, as the self-test is filled without any scientific guidance, one cannot rule out that participants interpreted the distinct motive categories differently. Furthermore, it must be kept in mind, that our findings stem from a (pre-)clinical sample of individuals who consider themselves at risk for detrimental gambling behaviour. Therefore, our effect estimates may not apply to other populations (Rudolph et al., 2023).

On the other hand, this study offers a more nuanced perspective on the association between gambling motives and transition towards more severe GD within an at-risk population. Given the primary purpose of the self-test (i.e., to encourage people who gamble to critically reflect on their behaviour) and its anonymous nature, the risk of social desirability bias is considered low. Second, we examined each transition point separately and did not collapse the distinct, unique motives by using domain-specific indices or factors scores. This enables a nuanced examination of associations and the identification of key motives within overarching motive domains. Last, we only accounted for data collected after the implementation of the German State Treaty on Gambling, which eased access to gambling activities by legalizing online gambling. This policy change might have contributed to a motivational shift for initiating and pursuing gambling engagement in both males and females who gamble.

## Conclusion

We conclude that the detrimental “effect” of distinct gambling motives is similar in males and females. Considering besides this “effect” gender-specific motive prevalence, financial motives (particularly in males) and mood-regulating escapist motives (particularly in females) warrant special attention. Hence, counselling and treatment programs for males and females who engage in detrimental gambling behaviour might prioritize the development of functional coping strategies and address cognitive distortions related to chasing losses and the illusion of control over monetary gains. Acknowledging furthermore the cross-gender relevance of enhancement motives at the transition point from no GD to GD, prevention and early intervention efforts might focus on raising awareness of “gambling” as a risky leisure activity.

## Supplementary Information

Below is the link to the electronic supplementary material.

Supplementary Material 1

Supplementary Material 2

Supplementary Material 3

Supplementary Material 4

## Data Availability

The data are property of the Bavarian Coordination Centre for Gambling Issues and are not publicly available. Data can be made available for research purposes upon reasonable request, subject to an individual data use agreement.
