# Supplementary material for: Associations Between Gambling Motives and More Severe Gambling Disorder – Does Gender Matter?
Source: J Gambl Stud. 2026 Feb 23;42(3):1207–21. doi: 10.1007/s10899-025-10470-3 (PMC13578037; doi:10.1007/s10899-025-10470-3)
Supplement: Supplementary file 1 [file 10899_2025_10470_MOESM1_ESM.docx]

|  | Male | Female |
| --- | --- | --- |
| Habit no GD (p=0.50) | -15.1724% | 12.5% |
| Habit Mild GD (p=0.16) | -43.0556% | 33.3333% |
| Habit Moderate GD (p=0.66) | -29.717% | 26.1538% |
| Habit Severe GD (p=0.73) | -52.9412% | 49.0196% |
| Fun no GD (p=0.83) | -71.4483% | 72.7941% |
| Fun Mild GD (p=0.38) | -68.6111% | 62.5% |
| Fun Moderate GD (p=0.14) | -66.9811% | 76.9231% |
| Fun Severe GD (p=0.05) | -68.3258% | 52.9412% |
| Thrill no GD (p=0.02) | -36.9655% | 25.7353% |
| Thrill Mild GD (p=0.66) | -63.3333% | 59.7222% |
| Thrill Moderate GD (p=0.76) | -52.1226% | 49.2308% |
| Thrill Severe GD (p=0.78) | -63.3484% | 66.6667% |
| Socializing Subclinical (p<0.001) | -2.75862% | 6.61765% |
| Socializing Mild GD (p=0.83) | -4.16667% | 5.55556% |
| Socializing Moderate GD (p=0.31) | -4.00943% | 7.69231% |
| Socializing Severe GD (p=0.27) | -10.8597% | 17.6471% |
| Gambling Friends no GD (p=0.22) | -19.5862% | 14.7059% |
| Gambling Friends Mild GD (p=0.01) | -25.5556% | 9.72222% |
| Gambling Friends Moderate GD (p=0.20) | -21.6981% | 13.8462% |
| Gambling Friends Severe GD (p=0.22) | -23.0769% | 17.6471% |
| Earn Money no GD (p<0.001) | -49.1034% | 32.3529% |
| Earn Money Mild GD (p=0.49) | -68.8889% | 63.8889% |
| Earn Money Moderate GD (p=0.89) | -61.7925% | 60% |
| Earn Money Severe GD (p=0.16) | -73.7557% | 62.7451% |
| Boredom no GD (p=0.18) | -38.8966% | 32.3529% |
| Boredom Mild GD (p=0.65) | -64.4444% | 68.0556% |
| Boredom Moderate GD (p=0.44) | -61.7925% | 67.6923% |
| Boredom Severe GD (p=0.06) | -67.8733% | 52.9412% |
| Stress no GD (p=0.02) | -10.2069% | 17.6471% |
| Stress Mild GD (p<0.001) | -43.6111% | 69.4444% |
| Stress Moderate GD (p=0.07) | -29.4811% | 41.5385% |
| Stress Severe GD (p=0.61) | -62.4434% | 68.6275% |
| Forget worries no GD (p<0.001) | -14.7586% | 26.4706% |
| Forget worries Mild GD (p<0.001) | -48.3333% | 75% |
| Forget worries Moderate GD (p=0.07) | -33.7264% | 46.1538% |
| Forget worries Severe GD (p=0.27) | -73.7557% | 82.3529% |
| When Depressed no GD (p<0.02) | -6.75862% | 13.2353% |
| When Depressed Mild GD (p<0.001) | -33.6111% | 56.9444% |
| When Depressed Moderate GD (p=0.03) | -19.5755% | 32.3077% |
| When Depressed Severe GD (p=0.84) | -59.7285% | 56.8627% |

GD=Gambling disorder

**Figure S1:** **Gambling motives among males and females at different severity levels of gambling disorder**
