# Supplementary material for: Associations Between Gambling Motives and More Severe Gambling Disorder – Does Gender Matter?
Source: J Gambl Stud. 2026 Feb 23;42(3):1207–21. doi: 10.1007/s10899-025-10470-3 (PMC13578037; doi:10.1007/s10899-025-10470-3)
Supplement: Supplementary file 2 [file 10899_2025_10470_MOESM2_ESM.docx]

**Table S1: Predictors for transition to higher severity levels of gambling disorder (including habit)**

|  | **Est.** | **SE** | **z value** | **p-value** | **OR** | **95 %- CI** | |
| --- | --- | --- | --- | --- | --- | --- | --- |
| *(Intercept): no GD* → *mild* | *-1.95* | *0.23* | *-8.49* | ***<0.001*** | *0.14* | *0.09* | *0.22* |
| *(Intercept): mild* → *moderate* | *-0.45* | *0.27* | *-1.63* | *0.10* | *0.64* | *0.38* | *1.09* |
| *(Intercept): moderate* → *severe* | *-1.26* | *0.34* | *-3.70* | ***<0.001*** | *0.29* | *0.15* | *0.55* |
| *Female: no GD* → *mild* | *-0.22* | *0.18* | *-1.19* | *0.24* | *0.81* | *0.56* | *1.15* |
| *Female: mild* → *moderate* | *0.28* | *0.20* | *1.43* | *0.15* | *1.33* | *0.90* | *1.96* |
| *Female: moderate* → *severe* | *0.01* | *0.22* | *0.05* | *0.96* | *1.01* | *0.66* | *1.54* |
| *Age: no GD* → *mild* | *-0.01* | *0.01* | *-0.98* | *0.33* | *1.00* | *0.99* | *1.01* |
| *Age: mild* → *moderate* | *-0.02* | *0.01* | *-3.18* | ***<0.001*** | *0.98* | *0.97* | *0.99* |
| *Age: moderate* → *severe* | *-0.01* | *0.01* | *-0.87* | *0.39* | *0.99* | *0.98* | *1.01* |
| Habit: no GD → mild | 0.57 | 0.15 | 3.84 | **<0.001** | 1.78 | 1.32 | 2.38 |
| Habit: mild → moderate | 0.44 | 0.15 | 3.00 | **<0.001** | 1.56 | 1.17 | 2.08 |
| Habit: moderate → severe | 0.35 | 0.17 | 2.10 | **0.04** | 1.42 | 1.02 | 1.97 |
| Thrill: no GD → mild | 0.44 | 0.13 | 3.48 | **<0.001** | 1.54 | 1.21 | 1.97 |
| Thrill: mild → moderate | 0.27 | 0.14 | 1.94 | **0.05** | 1.31 | 1.00 | 1.73 |
| Thrill: moderate → severe | -0.17 | 0.17 | -1.00 | 0.32 | 0.84 | 0.60 | 1.18 |
| Stress: no GD → mild | 0.83 | 0.18 | 4.72 | **<0.001** | 2.30 | 1.63 | 3.25 |
| Stress: mild → moderate | 0.33 | 0.17 | 1.90 | 0.06 | 1.39 | 0.99 | 1.94 |
| Stress: moderate->severe | 0.05 | 0.20 | 0.22 | 0.82 | 1.05 | 0.71 | 1.55 |
| Forget worries: no GD → mild | 0.62 | 0.15 | 4.00 | **<0.001** | 1.86 | 1.37 | 2.51 |
| Forget worries: mild → moderate | 0.47 | 0.16 | 2.97 | **<0.001** | 1.60 | 1.17 | 2.18 |
| Forget worries: moderate → severe | 0.85 | 0.20 | 4.38 | **<0.001** | 2.34 | 1.60 | 3.43 |
| Earn money: no GD → mild | 0.90 | 0.13 | 7.07 | **<0.001** | 2.45 | 1.91 | 3.14 |
| Earn money: mild → moderate | 0.42 | 0.15 | 2.90 | **<0.001** | 1.53 | 1.15 | 2.03 |
| Earn money: moderate → severe | 0.36 | 0.18 | 2.03 | **0.04** | 1.43 | 1.01 | 2.03 |
| Boredom: no GD → mild | 0.81 | 0.13 | 6.39 | **<0.001** | 2.25 | 1.75 | 2.89 |
| Boredom: mild → moderate | -0.19 | 0.15 | -1.27 | 0.20 | 0.83 | 0.62 | 1.11 |
| Boredom: moderate → severe | -0.27 | 0.18 | -1.56 | 0.12 | 0.76 | 0.54 | 1.07 |
| When depressed: no GD → mild | 0.52 | 0.20 | 2.61 | **0.01** | 1.68 | 1.14 | 2.49 |
| When depressed: mild → moderate | 0.47 | 0.18 | 2.63 | **0.01** | 1.60 | 1.13 | 2.27 |
| When depressed: moderate→ severe | 0.62 | 0.19 | 3.20 | **<0.001** | 1.85 | 1.27 | 2.70 |

bold values are significant;
CI = 95% confidence interval, OR = Odds Ratio, SE = Standard error
