# Supplementary material for: Associations Between Gambling Motives and More Severe Gambling Disorder – Does Gender Matter?
Source: J Gambl Stud. 2026 Feb 23;42(3):1207–21. doi: 10.1007/s10899-025-10470-3 (PMC13578037; doi:10.1007/s10899-025-10470-3)
Supplement: Supplementary file 3 [file 10899_2025_10470_MOESM3_ESM.docx]

**Table S2: Predictors for transition to higher severity levels of gambling disorder in males including habit**

|  | **Est.** | **SE** | **z value** | | **p-value** | | **OR** | | **95 %- CI** | | |
| --- | --- | --- | --- | --- | --- | --- | --- | --- | --- | --- | --- |
| *(Intercept): no GD → mild* | *-1.79* | *.25* | *-7.28* | ***<.001*** | | *.17* | | *.10* | | *.27* |  |
| *(Intercept): mild → moderate* | *-.42* | *.3* | *-1.43* | *.15* | | *.66* | | *.37* | | *1.17* |  |
| *(Intercept): moderate → severe* | *-1.63* | *.38* | *-4.28* | ***<.001*** | | *.2* | | *.09* | | *.41* |  |
| *Age: no GD → mild* | *-.01* | *.01* | *-1.09* | *.28* | | *.99* | | *.98* | | *1.00* |  |
| *Age: mild → moderate* | *-.02* | *.01* | *-2.83* | ***<.001*** | | *.98* | | *.97* | | *.99* |  |
| *Age: moderate → severe* | *.00* | *.01* | *-.05* | *.96* | | *1.00* | | *.98* | | *1.02* |  |
| Habit: no GD → mild | .60 | .16 | 3.74 | **<.001** | | 1.81 | | 1.33 | | 2.48 |  |
| Habit: mild → moderate | .50 | .16 | 3.14 | **<.001** | | 1.64 | | 1.21 | | 2.24 |  |
| Habit: moderate → severe | .25 | .19 | 1.34 | .18 | | 1.28 | | .89 | | 1.84 |  |
| Thrill: no GD → mild | .38 | .13 | 2.84 | **<.001** | | 1.46 | | 1.12 | | 1.9 |  |
| Thrill: mild → moderate | .29 | .15 | 1.87 | .06 | | 1.33 | | .99 | | 1.8 |  |
| Thrill: moderate → severe | -.21 | .19 | -1.08 | .28 | | .81 | | .56 | | 1.18 |  |
| Stress: no GD → mild | .86 | .19 | 4.43 | **<.001** | | 2.36 | | 1.62 | | 3.46 |  |
| Stress: mild → moderate | .29 | .19 | 1.53 | .13 | | 1.33 | | .92 | | 1.92 |  |
| Stress: moderate → severe | .10 | .22 | .47 | .64 | | 1.11 | | .72 | | 1.71 |  |
| Forget worries: no GD → mild | .65 | .17 | 3.86 | **<.001** | | 1.91 | | 1.37 | | 2.65 |  |
| Forget worries: mild → moderate | .39 | .17 | 2.28 | **.02** | | 1.47 | | 1.06 | | 2.05 |  |
| Forget worries: moderate → severe | .85 | .21 | 4.02 | **<.001** | | 2.33 | | 1.54 | | 3.52 |  |
| Earn money: no GD → mild | .78 | .14 | 5.73 | **<.001** | | 2.17 | | 1.67 | | 2.83 |  |
| Earn money: mild → moderate | .41 | .16 | 2.61 | **.01** | | 1.51 | | 1.11 | | 2.06 |  |
| Earn money: moderate → severe | .45 | .2 | 2.24 | **.03** | | 1.56 | | 1.06 | | 2.31 |  |
| Boredom: no GD → mild | .73 | .14 | 5.38 | **<.001** | | 2.08 | | 1.59 | | 2.72 |  |
| Boredom: mild → moderate | -.17 | .16 | -1.1 | .27 | | .84 | | .62 | | 1.15 |  |
| Boredom: moderate → severe | -.11 | .2 | -.57 | .57 | | .89 | | .61 | | 1.31 |  |
| When depressed: no GD → mild | .54 | .23 | 2.38 | **.02** | | 1.71 | | 1.10 | | 2.66 |  |
| When depressed: mild → moderate | .45 | .2 | 2.26 | **.02** | | 1.57 | | 1.06 | | 2.31 |  |
| When depressed: moderate → severe | .74 | .22 | 3.44 | **<.001** | | 2.09 | | 1.37 | | 3.19 |  |

bold values are significant;
CI = 95% confidence interval, OR = Odds Ratio, SE = Standard error
