# Supplementary material for: Associations Between Gambling Motives and More Severe Gambling Disorder – Does Gender Matter?
Source: J Gambl Stud. 2026 Feb 23;42(3):1207–21. doi: 10.1007/s10899-025-10470-3 (PMC13578037; doi:10.1007/s10899-025-10470-3)
Supplement: Supplementary file 4 [file 10899_2025_10470_MOESM4_ESM.docx]

**Table S3: Predictors for transition to higher severity levels of gambling disorder in females including habit**

|  | **Est.** | **SE** | **z value** | **p-value** | **OR** | **95 %- CI** | |
| --- | --- | --- | --- | --- | --- | --- | --- |
| *(Intercept): no GD → mild* | *-3.18* | *0.71* | *-4.51* | *<0.001* | *0.04* | *0.01* | *0.17* |
| *(Intercept): mild → moderate* | *-0.35* | *0.81* | *-0.44* | *0.66* | *0.70* | *0.14* | *3.43* |
| *(Intercept): moderate → severe* | *0.70* | *0.87* | *0.81* | *0.42* | *2.02* | *0.37* | *11.04* |
| *Age: no GD → mild* | *<0.001* | *0.01* | *0.05* | *0.96* | *1.00* | *0.97* | *1.03* |
| *Age: mild → moderate* | *-0.02* | *0.02* | *-1.44* | *0.15* | *0.98* | *0.95* | *1.01* |
| *Age: moderate → severe* | *-0.03* | *0.02* | *-1.8* | *0.07* | *0.97* | *0.94* | *1.00* |
| Habit: no GD → mild | 0.42 | 0.45 | 0.94 | 0.35 | 1.52 | 0.63 | 3.66 |
| Habit: mild → moderate | 0.1 | 0.41 | 0.24 | 0.81 | 1.10 | 0.49 | 2.48 |
| Habit: moderate → severe | 0.85 | 0.41 | 2.1 | **0.04** | 2.34 | 1.06 | 5.19 |
| Thrill: no GD → mild | 0.95 | 0.37 | 2.53 | **0.01** | 2.58 | 1.24 | 5.37 |
| Thrill: mild → moderate | -0.01 | 0.39 | -0.03 | 0.98 | 0.99 | 0.46 | 2.12 |
| Thrill: moderate → severe | 0.16 | 0.42 | 0.38 | 0.70 | 1.18 | 0.52 | 2.68 |
| Stress: no GD → mild | 0.75 | 0.45 | 1.66 | 0.10 | 2.11 | 0.87 | 5.12 |
| Stress: mild → moderate | 0.47 | 0.46 | 1.01 | 0.31 | 1.59 | 0.65 | 3.93 |
| Stress: moderate → severe | -0.24 | 0.51 | -0.47 | 0.64 | 0.78 | 0.29 | 2.14 |
| Forget: no GD → mild | 0.47 | 0.43 | 1.11 | 0.27 | 1.61 | 0.7 | 3.71 |
| Forget: mild → moderate | 1.13 | 0.47 | 2.42 | **0.02** | 3.08 | 1.24 | 7.69 |
| Forget: moderate → severe | 0.52 | 0.55 | 0.94 | 0.35 | 1.68 | 0.57 | 4.92 |
| Money: no GD → mild | 1.78 | 0.38 | 4.66 | **<0.001** | 5.94 | 2.81 | 12.56 |
| Money: mild → moderate | 0.48 | 0.39 | 1.23 | 0.22 | 1.62 | 0.75 | 3.50 |
| Money : moderate→ severe | -0.12 | 0.4 | -0.31 | 0.76 | 0.88 | 0.40 | 1.95 |
| Boredom: no GD → mild | 1.39 | 0.37 | 3.80 | **<0.001** | 4.02 | 1.96 | 8.24 |
| Boredom: mild → moderate | -0.32 | 0.41 | -0.79 | 0.43 | 0.73 | 0.33 | 1.61 |
| Boredom: moderate → severe | -0.89 | 0.41 | -2.14 | **0.03** | 0.41 | 0.18 | 0.93 |
| When depressed: no GD → mild | 0.45 | 0.45 | 1.00 | 0.32 | 1.57 | 0.65 | 3.77 |
| When depressed: mild → moderate | 0.65 | 0.42 | 1.54 | 0.12 | 1.92 | 0.84 | 4.4 |
| When depressed: moderate → severe | 0.05 | 0.45 | 0.12 | 0.91 | 1.05 | 0.44 | 2.54 |

bold values are significant;
CI = 95% confidence interval, OR = Odds Ratio, SE = Standard error
